# Refining Surface Copper Species on Cu/SiO_2_ Catalysts to Boost Furfural Hydrogenation to Furfuryl Alcohol

**DOI:** 10.3390/molecules30020225

**Published:** 2025-01-08

**Authors:** Jieqiong Wang, Jingyi Yang, Zezheng Bing, Yuanyuan Gao, Tao Yang, Qiaoyun Liu, Meng Zhang, Zhongyi Liu

**Affiliations:** 1 College of Chemistry, Zhengzhou University, Zhengzhou 450001, China; 2 School of Chemical Engineering, Zhengzhou University, Zhengzhou 450001, China; 3 State Key Laboratory of Coking Coal Resources Green Exploitation, Zhengzhou University, Zhengzhou 450001, China

**Keywords:** layered copper silicate, Cu^+^/(Cu^0^+Cu^+^), metal–support interaction, selective hydrogenation of furfural, activation of C=O

## Abstract

Controllable hydrogenation of carbonyl groups (C=O) is crucial for converting furfural into high-value furfuryl alcohol. Instead of traditional impregnation method, a novel Cu-based catalyst (Cu/SiO_2_) is prepared using the ammonia evaporation method (AE) for the efficient hydrogenation of furfural to furfuryl alcohol under mild conditions. At the reaction conditions of 90 °C and 1 MPa H_2_, the 5Cu/SiO_2_-AE sample showed optimal performance with higher turnover frequency (36.0 h^−1^) and furfuryl alcohol selectivity (>99.9%). After five cycles, the catalyst recycled still showed a high reaction activity and selectivity for furfuryl alcohol. Characterization results such as XRD, H_2_-TPR, FT-IR, and XPS showed that the excellent catalytic performance of 5Cu/SiO_2_-AE catalyst was attributed to the formation of layered copper silicate and the high dispersion of Cu species. Furthermore, the formation of layered copper silicate resulted in a higher ratio of Cu^+^/(Cu^0^+Cu^+^) at a reduction temperature of 250 °C, which was also responsible for the optimum activity. This work showed the importance of controllable synthesis of layered copper silicate in improving the catalytic performance of copper-containing catalyst.

## 1. Introduction

As non-renewable fossil resources are depleted and environmental pollution worsens, biomass becomes one of the most important renewable resources which has been utilized for the green synthesis of fossil fuels and fine chemicals [1,2,3]. Among them, the hydrogenation of unsaturated compounds containing C=O bonds is critically important [4], especially in pharmaceutical, flavor, and agrochemical manufacturing. Furfural (FAL), a biomass platform molecule, links biomass with a range of multifunctional chemicals [5,6]. Its selective hydrogenation product of furfuryl alcohol (FOL) is widely used in the production of dark thermostatic resins [7], fibers, rubbers, and fuel additives with high added value [8,9]. However, FAL is a polyfunctional chemical consisting of functional groups (C=C, C=O, and furan heterocycles), leading to a variety of hydrogenation products [10,11]. The bond energy of C=C (615 kJ·mol^−1^) is lower than that of the C=O bond (715 kJ·mol^−1^) [12], resulting in a tendency for FAL to undergo excessive hydrogenation and exhibit low selectivity towards FOL. Therefore, the development of an efficient catalyst for the selective hydrogenation of FAL to FOL is necessary [13,14,15].

Copper chromate (CrCuO_4_) is currently used as an industrial catalyst for the hydrogenation of FAL to FOL [16], but the presence of chromium species (Cr(VI) and Cr(V)) and harsh reaction conditions drive interest in developing more energy efficient and environmentally friendly catalysts [17]. Noble metals such as Pt [18,19], Pd [20,21], and Ru [22,23] are known for their catalytic efficiency but are limited in large-scale applications due to their high preparation cost and the formation of by-products during the reactions. In recent years, non-noble Cu catalysts such as Cu/SiO_2_ [24,25], Cu/CeO_2_ [26,27], Cu/MgO [28], and Cu/ZnO [29] catalysts have been widely used for the selective hydrogenation of FAL. This is attributed to their specific hydrogenation selectivity of the C=O bond. Among them, Cu/SiO_2_ catalysts are commonly employed because of their high selectivity due to their strong metal–support interaction, which facilitates the construction of copper active sites [30].

Previous studies by Gong and Yue et al. demonstrated that the synergistic effect between Cu^+^ and Cu^0^ was the reason for the well hydrogenation reaction of Cu/SiO_2_, where Cu^+^ acted as the primary hydrogenation active site, and Cu^0^ facilitated the transfer of H atoms [31,32,33]. Baker et al. reported that the double site composed of Cu^0^ and Cu^+^ acted as the rate-determining step of the bimolecular surface reaction of FAL and H_2_ [34]. Ghambarian et al. proved that Cu^0^ in Cu/MgO catalysts served as the active site, which promoted the occurrence for the hydrogenation of FAL [35]. Yang et al. demonstrated that the layered copper silicate facilitated the well dispersion of Cu^+^, which functioned as the active site, but the regulation of the formation of layered copper silicate had not been well investigated [36]. Nevertheless, the identity of the active sites in Cu-based catalysts had been a topic of significant debate in FAL reactions. The relative amounts of Cu^0^, Cu^+^, and Cu^2+^ were influenced by the preparation methods as well as the pretreatment conditions. The different reduction performances of layered copper silicate and CuO should also be carefully studied [37,38,39].

In this work, a series of low-load (5 wt%) Cu/SiO_2_ catalysts were prepared via the evaporated ammonia and impregnation method and were used in the selective hydrogenation of FAL to FOL at different reaction conditions. The physicochemical properties of the catalysts were carefully studied to investigated the effect of the preparation methods and thermal treatment, thus controlling the compounds of active sites. The active copper species was recognized to establish the relevance between catalytic performance and active site, allowing for the investigate of the reaction mechanism and the role of the active sites [40,41,42,43,44,45]. This work provided a new idea to regulate the active sites of Cu, thus improving the performance of C=O hydrogenation.

## 2. Result and Discussion

### 2.1. Textural Properties of Catalysts

The Cu/SiO_2_ catalysts were prepared using the ammonia evaporation (AE) method and incipient wetness impregnation (IWI) method via the mass loading of Cu at 5%. The copper content in 5Cu/SiO_2_-IWI and Cu/SiO_2_-AE was determined using ICP-OES, and the actual copper content closely matched the theoretical values, confirming that the copper was completely loaded onto the support. N_2_ adsorption–desorption isotherms and pore size distribution of all catalysts showed a typical type IV Langmuir adsorption isotherm with an H3-type hysteresis loop (Figures S1 and S2), corresponding to a mesoporous structure [46]. As depicted in Table 1, the specific surface area (S_BET_) of the catalysts significantly diminished after metal loading compared to the support, confirming the effective anchoring of Cu nanoparticles (NPs). The copper surface areas (S_Cu_) and dispersion (D_Cu_) of all the samples are listed in Table 1 according to the calculation from the N_2_O titration [47]. The S_Cu_ and D_Cu_ of Cu/SiO_2_-AE catalyst increased with increasing calcination temperature but subsequently decreased, and the catalysts prepared using AE displayed a significantly higher dispersion of Cu than that prepared via IWI. The 5Cu/SiO_2_-AE catalyst reached a maximum value of S_Cu_ (4.74 m^2^_Cu_/g_cat_) and D_Cu_ (27.94%). In contrast, 5Cu/SiO_2_-AE-C500 showed lower S_Cu_ (3.22 m^2^_Cu_/g_cat_) and D_Cu_ (21.34%) than the other catalysts, which might be ascribed to the destruction of the interaction between Cu and SiO_2_.

**Figure 1:**
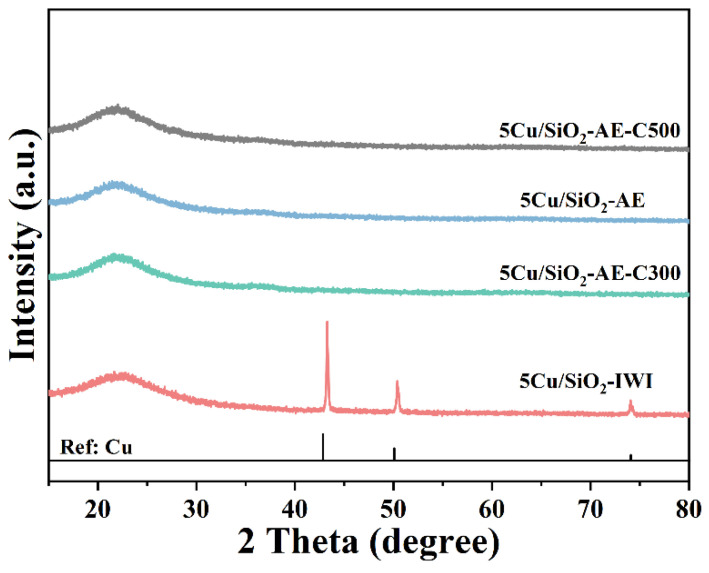
XRD patterns of Cu/SiO_2_ catalysts.

X-ray diffraction (XRD) patterns were employed to analyze the crystal structures of catalysts. The sharp diffraction peaks of 5Cu/SiO_2_-IWI at 42.8°, 50.4°, and 74.1° corresponded to the Cu (111), (200), and (220) planes, respectively (Figure 1) [48], which suggested the formation of larger Cu NPs on the surface of the catalyst. In contrast, the XRD pattern of Cu/SiO_2_-AE showed no distinct diffraction peaks for the Cu species (Figure S3) excepted an amorphous silica peak at 22° [49,50], suggesting that the copper in the catalysts prepared via the AE method was highly dispersed on the support. In conclusion, the catalysts prepared using AE showed a higher dispersion of Cu.

**Figure 2:**
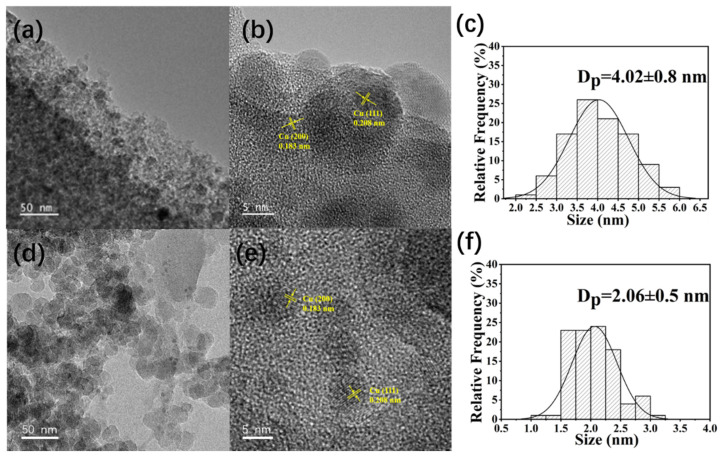
TEM images of catalysts: (**a**–**c**) 5Cu/SiO_2_-IWI and (**d**–**f**) 5Cu/SiO_2_-AE.

Figure 2 shows TEM images of the 5Cu/SiO_2_-IWI and 5Cu/SiO_2_-AE catalysts after reduction at 250 °C. The lattice fringe spacing measured in HRTEM images was 0.208 nm and 0.183 nm, corresponding to the (111) and (200) crystal faces of Cu, respectively. The 5Cu/SiO_2_-IWI catalyst showed some large Cu NPs, and the average pore diameter was about 4 nm, confirming the uncontrollable synthesis of nanoparticles via IWI. However, for 5Cu/SiO_2_-AE, Cu NPs were uniformly dispersed with a diameter of 2.06 nm, without apparent aggregation. This observation highlighted the effectiveness of the AE method on enhancing metal dispersion, which corresponded well with the XRD results. However, an increase in the size of the Cu NPs was observed in 5Cu/SiO_2_-AE-R300 and 5Cu/SiO_2_-AE-R350 (Figure S4). This was attributed to the low Tammann temperature of Cu, which facilitated atomic migration within the lattice at high temperatures, leading to the aggregation and sintering of Cu nanoparticles [51].

### 2.2. Physicochemical Properties of the Catalysts

#### 2.2.1. H_2_-TPR

**Figure 3:**
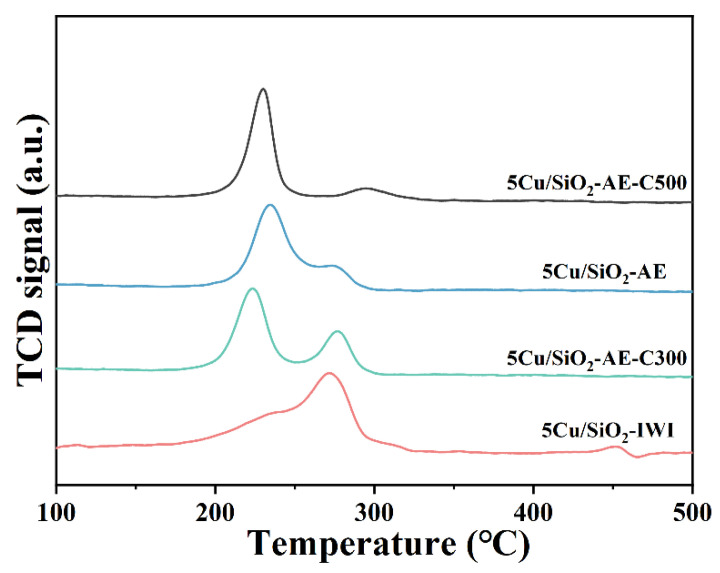
H_2_-TPR profiles of 5Cu/SiO_2_-IWI and Cu/SiO_2_-AE at different calcination temperatures.

The H_2_-TPR profiles of catalysts are shown in Figure 3; the reduction peaks were mainly observed between 200 and 300 °C. The nearly symmetrical reduction peaks for Cu/SiO_2_-AE between 209 and 240 °C could be attributed to the reduction in layered copper silicate to Cu^+^ and the direct conversion of well-dispersed Cu species to Cu^0^ [52,53]. Meanwhile, the reduction peak at 275 °C in all catalysts indicated that there might be bulk copper species on the surface of the catalyst, which was difficult to be reduced [54]. It was clear that the cooper species was more easily reduced via the AE method, which might be ascribed to the formation of the highly dispersed copper species. Thus, the AE method could be effectively used to achieve good dispersion of Cu species on the support, which would facilitate the formation of smaller Cu NPs to provide more active sites.

#### 2.2.2. XPS

**Figure 4:**
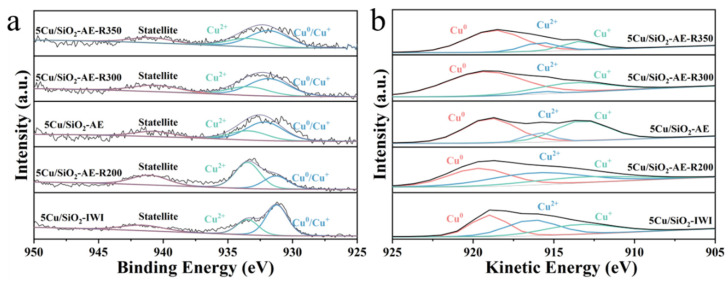
(**a**) Cu 2p XPS and (**b**) Cu LMM XAES spectra of 5Cu/SiO_2_-IWI and Cu/SiO_2_-AE at different reduction temperatures.

The surface composition and oxidation states of the catalysts were investigated via XPS Cu 2p and Cu LMM AES spectra (Figure 4 and Figure S5). The peaks at 932.2 and 933.8 eV are shown in Figure 4a and Figure S5a and were attributed to the low oxidation state species (Cu^0^/Cu^+^) and Cu^2+^ species, respectively [55,56]. Satellite peak at 941.6 eV provided evidence of Cu^2+^, indicating that part of Cu^2+^ had been reduced to Cu^0^ and Cu^+^ [33], which was in agreement with the TPR analyses.

To distinguish between Cu^0^ and Cu^+^, the Auger spectrum was employed to differentiate between Cu^0^ and Cu^+^ species. The deconvolution analysis of Figure 4b and Figure S5b showed three peaks of 918.4, 914.2, and 915.6 eV, corresponding to Cu^0^, Cu^+^, and Cu^2+^, respectively [37]. The ratio of Cu^0^ and Cu^+^ species on the support surface was calculated. As shown in Table S1, the catalysts prepared using IWI and AE methods contained the same proportion of (Cu^0^+Cu^+^)/Cu. At a higher calcination temperature (500 °C) and a lower reduction temperature (200 °C), the number of active sites (Cu^0^+Cu^+^) on the catalyst surface was lower due to high-temperature sintering and an incomplete reduction, respectively. As the reduction temperature increased, the Cu^+^/(Cu^0^+Cu^+^) ratio exhibited a volcano-shaped distribution, with 5Cu/SiO_2_-AE reaching the highest value of 45.4%. Meanwhile, the Cu^0^/(Cu^0^+Cu^+^) ratio increased with higher reduction temperatures, with 5Cu/SiO_2_-AE-R350 achieving the highest value of 80.8%.

#### 2.2.3. FT-IR

**Figure 5:**
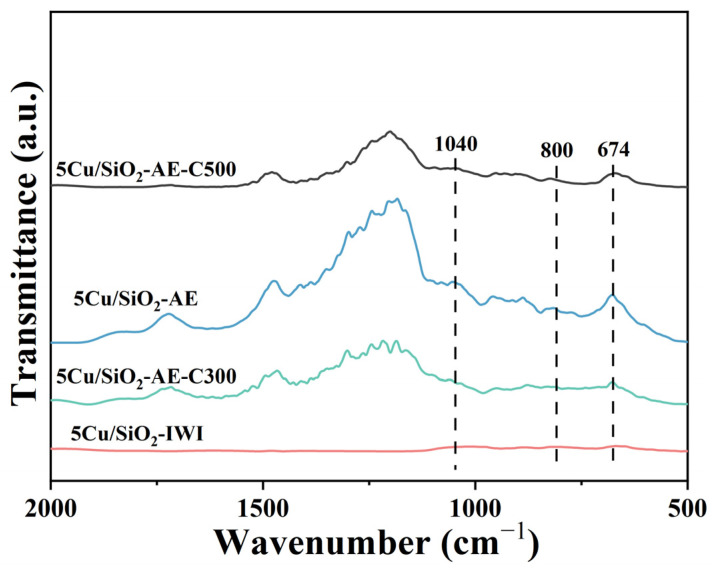
FT-IR spectra of 5Cu/SiO_2_-IWI and Cu/SiO_2_-AE at different calcination temperatures.

FT-IR measurements of 5Cu/SiO_2_-IWI and Cu/SiO_2_-AE at different calcination temperatures revealed an absorption band for the carboxyl groups near 1710 cm^−1^, primarily due to water absorption on the catalyst surface (Figure 5). The presence of ν(Si-O) and δ(-OH) bands for layered copper silicate at 1040 and 674 cm^−1^ was present in Cu/SiO_2_-AE [41] but not in 5Cu/SiO_2_-IWI, indicating that the AE method enhanced the formation of layered copper silicate.

The ratio of I_674_/I_800_ was calculated to reveal the contents of layered copper silicate [57]. As shown in Table S2, the highest I_674_/I_800_ value of 5Cu/SiO_2_-AE (0.19) indicated a substantial amount of layered copper silicate and a strong metal–support interaction between copper and SiO_2_, which promoted the formation of Cu^+^ species. As the calcination temperature increased to 500 °C, the contents of the layered copper silicate decreased significantly, likely due to the collapse of its structure and the agglomeration of Cu species at high temperatures. This also led to a decrease in Cu dispersion and surface area [40], consistent with the N_2_O chemisorption results. Based on the comprehensive results from TEM, XPS, and FT-IR, the catalyst prepared using the AE method formed layered copper silicate, which facilitated the generation of Cu^+^ during the reduction process and a higher dispersion of Cu NPs on the support surface.

### 2.3. Catalyst Performance for FAL Hydrogenation

In order to evaluate the catalytic activity and stability of 5Cu/SiO_2_-IWI and Cu/SiO_2_-AE catalysts in FAL hydrogenation, the effects of reaction conditions (reaction temperature, pressure, time, and catalyst stability) were investigated.

#### 2.3.1. Effects of Reaction Conditions

**Figure 6:**
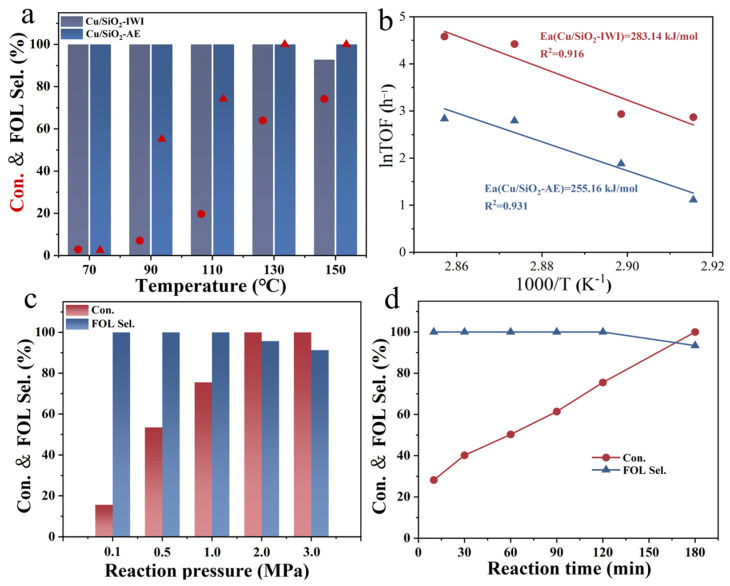
Effect of (**a**) reaction temperature and (**b**) the Arrhenius regressions in 5Cu/SiO_2_-IWI and 5Cu/SiO_2_-AE, as well as (**c**) reaction pressure and (**d**) reaction time in 5Cu/SiO_2_-AE. Reaction conditions: (**a**) P_(H2)_ = 1 MPa, t = 2 h; (**c**) T = 110 °C, t = 2 h; (**d**) T = 110 °C, and P_(H2)_ = 1 MPa.

Reaction temperature was a key factor affecting the activity and selectivity of the catalysts (Figure 6a). For 5Cu/SiO_2_-AE, FAL conversion increased with increasing reaction temperature, exceeding 99.9% at 130 °C, and FOL selectivity was kept above 99.9%. In contrast, 5Cu/SiO_2_-IWI showed a lower reaction activity, and the FAL conversion reached its highest (74.2%) at 150 °C, while FOL selectivity decreased to 92.8%. It was likely due to the polymerization of the catalyst into difurfuryl ether, which might cover the active site, thus leading to the decreased activity. As shown in Figure 6c, the FAL conversion increased with rising H_2_ pressure, reaching 99.9% at 2 MPa, while the selectivity for FOL decreased to 97.1%. In Figure 6d, the conversion of FAL increased with the reaction time, reaching 99.9% at 3h, but the selectivity for FOL decreased to 95.3%. Hence, it could be determined that the optimal reaction conditions were 130 °C, at 1 MPa H_2_, and for 2 h.

Furthermore, control experiments with the optimal 5Cu/SiO_2_-AE catalyst under N_2_ instead of H_2_ conditions showed that the catalyst was nearly inactive for FAL hydrogenation (Table 2, entry 1). This confirmed that the H_2_ was the sole hydrogen donor for FAL selective hydrogenation [58].

#### 2.3.2. Catalytic Stability

The hydrogenation stability of 5Cu/SiO_2_-AE catalyst was crucial in the hydrogenation of FAL to FOL. After five cycles, the catalyst still remained, with a FAL conversion of 55.8% and a selectivity of FOL over 99.9% (Figure S6a), indicating excellent stability. Compared to the fresh catalyst, the TGA curve (Figure S6b) and TEM images (Figure S7) showed no significant changes. This suggested that there was no notable weight loss in the spent catalyst, and its structural integrity remained stable. Therefore, 5Cu/SiO_2_-AE not only exhibited the highest FAL conversion and best FOL selectivity among the catalysts but also demonstrated outstanding thermal cycling stability.

### 2.4. Mechanism of FAL Hydrogenation on Catalysts

As listed in Table 2, the effects of calcination (Table 2, entries 2–4) and reduction temperatures (Table 2, entries 5–7) on the catalytic performance were evaluated. At 90 °C, the performance of the catalysts exhibited a dramatic change with increasing calcination and reduction temperatures. The 5Cu/SiO_2_-AE showed the highest catalytic performance and turnover frequency (36.0 h^−1^). Specifically, 5Cu/SiO_2_-AE-C500 exhibited a drastic decrease in activity; this could be attributed to a severe collapse of the layered copper silicate structure, resulting in the reduction in active sites and the agglomeration of Cu NPs.

**Figure 7:**
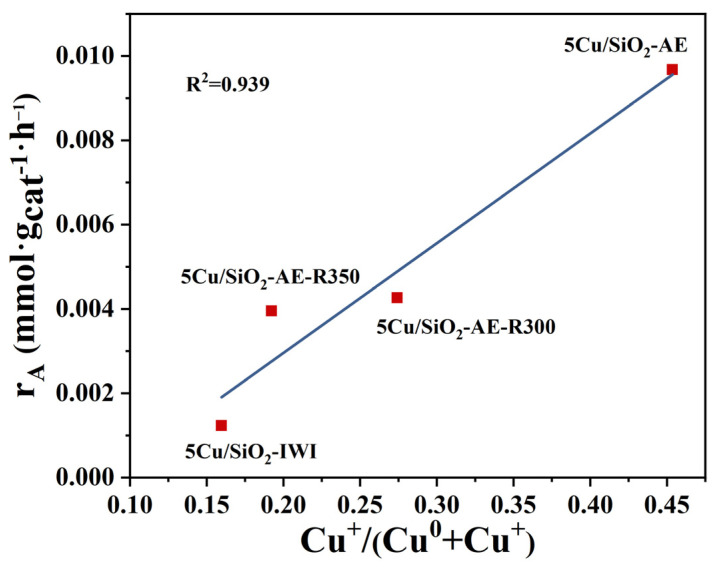
Cu^+^/(Cu^0^+Cu^+^) ratio versus the reduction temperature at 90 °C.

By integrating the activity data and XPS results, the relationship between the reaction rate (r_A_) and the Cu^+^/(Cu^0^+Cu^+^) ratio was studied to establish a structure–activity relationship. As shown in Figure 7, r_A_ exhibited a strong linear correlation with surface Cu^+^ content, suggesting that Cu^+^ played a critical role in the FAL hydrogenation reaction. Moreover, Cu^+^ had been reported to function as an electrophilic or Lewis acid site, adsorbing and polarizing the electron-rich C=O bond in furfural, thereby enhancing the selectivity for FOL hydrogenation [59]. Additionally, the FAL hydrogenation reaction was considered a pseudo first-order reaction [60]. By controlling the conversion within 20%, the apparent activation energy (E_a_) was calculated using the Arrhenius equation. The E_a_ values for 5Cu/SiO_2_-AE and 5Cu/SiO_2_-IWI were determined to be 255.16 kJ·mol^−1^ and 283.14 kJ·mol^−1^ (Figure 6b), respectively, indicating that 5Cu/SiO_2_-AE prepared via the AE method provided a low-energy barrier pathway, resulting in a high FAL conversion rate.

**Figure 8:**
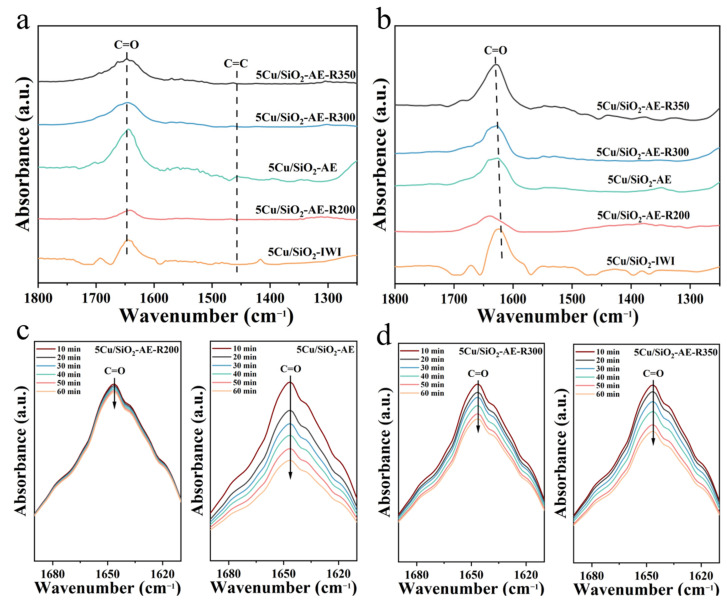
DRIFT spectra of FAL (**a**) adsorption at 30 °C; (**b**) desorption at 90 °C; and (**c**,**d**) desorption via a temperature program on the catalysts.

In order to gain further insights into the adsorption and activation of FAL with the catalysts, an in situ FT-IR spectroscopic technology was employed. As depicted in Figure 8a, the peaks at 1600–1700 cm^−1^ was assigned to the stretching vibrations of C=O bonds of the adsorbed FAL [61,62,63], while the peak at 1494 cm^−1^ corresponded to the C=C of FAL but was not obvious [64,65]. This indicated that FAL was adsorbed on the catalyst surface through C=O by η1; thus, the hydrogenation by-products involving furan ring hydrogenation were inhibited during the FAL hydrogenation process, consistent with the fact that the Cu/SiO_2_-AE catalysts achieved a 99.9% selectivity for FOL [66]. As the temperature increased (Figures S8 and S9), the original C=O, C-C, and C=C bonds gradually weakened, indicating that the physisorbed FAL desorbed from the catalyst surface with rising temperature [67]. The chemisorbed FAL remained after purging with N_2_ at 90 °C. Notably, the similar ν(C=O) adsorption peak for both Cu/SiO_2_-AE and 5Cu/SiO_2_-IWI at 1650 cm^−1^ (Figure 8b) might be ascribed to the adsorption of C=O on Cu^0^. For 5Cu/SiO_2_-AE, the blue shift in the ν(C=O) adsorption peak indicated that the lower adsorption strength might be ascribed to the existence of Cu^+^. Combining the catalytic performances, the weaker adsorption of C=O on Cu^+^ was favorable for the selective hydrogenation of carbonyl in FAL. Upon introducing H_2_ at 90 °C (Figure 8c,d), 5Cu/SiO_2_-AE showed a faster decrease in the C=O characteristic band at 1648 cm^−1^, indicating a higher catalytic activity, whereas the change in 5Cu/SiO_2_-IWI (Figure S10) was minimal, which was consistent with the superior activity of 5Cu/SiO_2_-AE in the hydrogenation of FAL.

Based on the previous discussions on evaluation and characterization, we found that the Cu/SiO_2_ catalysts had a stronger affinity for C=O adsorption rather than C=C, which effectively prevented the formation of by-products through the furan ring hydrogenation, leading to their high selectivity for FOL. Mechanistic studies utilizing in situ FT-IR revealed that 5Cu/SiO_2_-AE exhibited a higher FAL conversion rate and suitable adsorption strength for C=O, which might be beneficial for the hydrogenation of C=O.

## 3. Experimental Section

### 3.1. Catalyst Materials

Silica sol (SiO_2_, 30%, Macklin, Shanghai, China) and high-purity fumed silica (SiO_2_, Aladdin, Shanghai, China) with a specific surface area of 480 m^2^/g were used as supports. The active metal precursor was copper nitrate trihydrate (Cu(NO_3_)_2_·3H_2_O, 99%, Macklin, Shanghai, China), and the buffer used was urea (CH_4_N_2_O, 99%, Aladdin, Shanghai, China). The pH value during the catalyst preparation was regulated using aqueous ammonia (25 wt%, Aladdin, Shanghai, China). All chemicals utilized were analytical grade.

### 3.2. Catalyst Preparation

The Cu/SiO_2_ (5 wt% Cu) catalyst was prepared using the ammonia evaporation (AE) method, referred to as Cu/SiO_2_-AE, as described below. Briefly, 2.36 mmol of Cu(NO_3_)_2_·3H_2_O and 2 g of CH_4_N_2_O were dissolved in 100 mL of deionized water, followed by the addition of an ammonia aqueous solution. The mixed solution was stirred for 1 h with a pH value of 12. A total of 10 g of silica gel was subsequently added into the copper ammonia solution and stirred at 30 °C for 1 h. Ammonia evaporation was carried out by boiling the suspension at 80 °C until the pH value decreased to 6–7 and then washed with deionized water and dried overnight at 60 °C. Then, the catalyst precursor was calcined in air at 400 °C for 2 h and reduced at 250 °C for 2 h in a 10 vol% H_2_/Ar atmosphere, denoted as 5Cu/SiO_2_-AE. In addition, the catalyst was calcined in air at different temperatures and with the same reduction temperature (250 °C), donated as 5Cu/SiO_2_-AE-CX_1_ (X_1_ = 300, 500 °C); the catalyst was calcined at the same temperature (400 °C) but reduced at different temperatures, donated as 5Cu/SiO_2_-AE-RX_2_ (X_2_ = 200, 300, 350 °C).

For comparison, Cu/SiO_2_ was synthesized using the traditional incipient wetness impregnation (IWI) method. In brief, 2.36 mmol of Cu(NO_3_)_2_·3H_2_O was dissolved in 1 mL of deionized water. A total of 3.0 g of silica sol was subsequently added, and the mixture was stirred overnight at room temperature, and then dried at 60 °C overnight. The catalyst precursor was calcined at 400 °C for 2 h in air and reduced at 250 °C for 2 h in a 10 vol% H_2_/Ar atmosphere, denoted as 5Cu/SiO_2_-IWI.

### 3.3. Catalyst Characterization

The actual Cu loading was determined via inductively coupled plasma optical emission spectroscopy (ICP-OES) on Shimadzu ICPE-9820 (Micromeritics, Norcross, GA, USA). Powder X-ray diffraction (XRD) patterns were recorded on a SmartLab SE diffractometer at a scan speed of 0.1°/s over the 2θ range of 10–90°. The specific surface area of the samples was calculated using the Brunauer–Emmett–Teller (BET) method, performed on a Micromeritics ASAP 2420 instrument at −196 °C. Before the measurement, the samples were pretreated via heating at 150 °C for 6 h. The pore size distribution was determined using the Barrett–Joyner–Halenda (BJH) method using N_2_ adsorption data. A thermogravimetric analysis (TGA) was carried out on a STA2500 synchronous thermal analyzer from 50 to 850 °C under air (10 °C·min^−1^). The morphology of the catalysts was characterized using transmission electron microscope (TEM, JEOL-2100F, 200 kV, Japan). X-ray photoelectron spectroscopy (XPS) and X-ray-excited Auger electron spectroscopy (XAES) were conducted with an AXIS SUPRA spectrometer (Al Kα hν = 1486.6 eV). To avoid reoxidation during XPS tests, the samples were sealed in the sample bottle with full N_2_ and fixed in the XPS vacuum chamber within 10 min after reduction. C1s (284.8 eV) was referenced as an internal standard to calibrate all binding energy (BE) values.

The temperature-programmed reduction using H_2_ (H_2_-TPR) and N_2_O chemisorption (N_2_O-TPR) were performed using a Quanrachrome Autosorb-IQ gas adsorption analyzer (Micromeritics, Norcross, GA, USA). For the H_2_-TPR test, the catalyst (100 mg) was pretreated in Ar (50 mL·min^−1^) at 150 °C for 40 min and cooled to 50 °C. Next, 10 vol% H_2_/Ar (30 mL·min^−1^) was purged for 20 min to stabilize the baseline, as monitored by a thermal conductivity detector (TCD, Micromeritics, Norcross, GA, USA). The reduction profile was then recorded from 40 °C to 800 °C at a heating rate of 10 °C/min. A N_2_O titration was conducted to measure the surface area of metallic copper (S_Cu_) and copper dispersion (D_Cu_). About 100 mg of the sample was pretreated in He atmosphere for 0.5 h at 150 °C, cooled to 50 °C, and then switched to a 10 vol% H_2_/Ar mixture to reduce the catalyst for 1 h at different reduction temperatures (200, 250, 300, and 350 °C). The H_2_ consumption amount was recorded as A_1_. Next, the sample was cooled to 50 °C in He and then exposed to 10 vol% N_2_O/He for 2 h, ensuring that the surface of the metallic copper was oxidized to Cu_2_O. After purged for 0.5 h with He, the sample was reduced again in 10 vol%H_2_/Ar at the same reduction temperature for 1 h, and the H_2_ consumption amount was recorded as A_2_. The dispersion and the surface area of Cu were calculated using Equations (1) and (2):

(1) $$D_{\mathrm{Cu}}=\left[2A_{2}/A_{1}\right]\times 100\%$$

(2) $$S_{\mathrm{Cu}}=(2\times N_{A}\times A_{2})/(3286\times 1.4\times 10^{19}{\times m}_{\mathrm{cat}.})$$

In situ diffuse reflectance infrared Fourier transform spectroscopy (in situ FT-IR) was conducted on the prepared catalysts with an INVENIO S Fourier infrared spectrometer from Bruker, Germany. Firstly, catalysts at different reduction temperatures were reduced with 10 vol.% H_2_/Ar (30 mL/min) for 1 h. After the temperature was lowered to 30 °C, the background spectra were collected. Furfural was then introduced into the cuvette with a bubbler using N_2_ (10 mL/min) as the carrier gas. Infrared spectra were collected every 10 min for 1 h of adsorption. The spectra were collected when the adsorption was stabilized. Subsequently, N_2_ was introduced to elevate the temperature to 90 °C, and the desorption stability spectra were recorded. Upon completion of the desorption process, a 10 vol.% H_2_/Ar mixture (30 mL/min) was introduced for in situ hydrogenation. Spectra were then collected every 10 min for 1 h.

### 3.4. Catalytic Performance Test

The selective hydrogenation of furfural (FAL) was conducted in a 100 mL stainless steel high-pressure reactor (YZQR-100, Shanghai Yanzheng, China) equipped with a liquid phase autosampler. FAL (1 g, Macklin, 99%) was mixed in 30 mL of isopropanol (Macklin, 99.5%) in the presence of the catalyst (0.3 g) and poured into the reactor. After purging with N_2_, the reactor was pressurized to 1 MPa with H_2_, heated to the reaction temperature, and then maintained at 800 rpm. For the recyclability test, the catalyst was washed six times with isopropanol and any loss of catalyst was compensated for in subsequent runs. Upon completion of the reaction, the liquid product was collected and filtered through a 0.2 μm membrane filter to obtain a particle-free solution before further analysis. The liquid product was analyzed via gas chromatography (GC) using a flame ionization detector (FID, 8890-5977B GC-MS, Agilent, Santa Clara, CA, USA) with the SH-RXI-5SIL capillary column (30 m, 0.25 mm). The conversion rates (Con.) and FOL selectivity yields (Sel.) were measured according to Equations (3) and (4) as follows:

(3) $$\mathrm{Con}.\;(\%)=\frac{C_{0.\mathrm{FAL}}-C_{t.\mathrm{FAL}}}{C_{0.\mathrm{FAL}}}\times 100\%$$

(4) $$\mathrm{Sel}.\;(\%)\;=\frac{C_{\mathrm{FOL}}}{C_{0.\mathrm{FAL}}-C_{t.\mathrm{FAL}}}\times 100\%$$

where C_0.FAL_ and C_t.FAL_ represent the molar concentration of FAL at the initial time and at the time of interest, respectively. C_FOL_ signifies the molar concentration of the generated FOL.

In a typical cyclic test, the solid catalyst was centrifuged at 7000× *g* rpm and washed five times with cyclohexane. After overnight drying at 65 °C, the catalyst was directly used for the subsequent reaction.

The turnover frequency (TOF), which represented the number of FAL transformations per active site per hour, was calculated based on surface Cu atoms using Equation (5):

(5) $$\mathrm{TOF}=\frac{\gamma_{t}\times M_{\mathrm{Cu}}}{W_{\mathrm{Cu}}\times D_{\mathrm{Cu}}}$$

where γ_t_ is the reaction rate (mol·g^−1^·h^−1^) at low conversion (<10%), corresponding to the FOL conversion per gram of catalyst per hour. M_Cu_ and W_Cu_ are the molar mass (g·mol^−1^) and loading (wt%) of Cu, respectively. The copper dispersion D_Cu_ (%) is determined via N_2_O chemisorption.

The preparation of FOL via the hydrogenation of FAL could be regarded as a pseudo-primary kinetic reaction due to the significant excess of H_2_ in the reaction process. Based on the previous literature, Equations (6) and (7) could be used to study chemical kinetics:

(6) $$C_{0}-C_{t}=C_{0}(1-e^{-\mathrm{kt}})$$

(7) $$\mathrm{lnk}=-\frac{E_{a}}{\mathrm{RT}}+\mathrm{lnA}$$

where k denotes the empirical kinetic constant; Ea denotes the apparent activation energy; T denotes the temperature at which the reaction proceeds; R denotes the molar gas constant, and A denotes the pre-factor.

## 4. Conclusions

In conclusion, compared to the IWI method, the 5Cu/SiO_2_-AE catalyst prepared via the AE method demonstrated superior activity and a low activation energy. The formation of layered copper silicate through the AE method was important for enhancing catalytic performance, which facilitated the generation of Cu^+^ species and promoted the dispersion of Cu NPs. The correlation between catalytic activity and the Cu^+^/(Cu^0^+Cu^+^) ratio suggested that Cu^+^ played a crucial role in the reaction, promoting the activation of C=O and accelerating the conversion of FAL. Moreover, 5Cu/SiO_2_-AE exhibited excellent stability even after five cycles. This work provides valuable insights into the selective hydrogenation of C=O in FAL by controlling the formation of layered copper silicate and contributes to the efficient utilization of biomass resources and the development of green energy.

## Figures and Tables

**Table 1:** Textural properties of catalysts.

Sample	S_BET_ *^a^* (m^2^/g)	V_total_ (cm^3^/g)	d_p_ *^b^* (nm)	Cu Loading *^c^* (wt%)	D_Cu_ *^d^* (%)	S_Cu_ *^e^* (m^2^_Cu_/g_cat_)
SiO_2_	474	0.75	5.2	-	-	-
5Cu/SiO_2_-IWI	401	0.49	6.4	4.57	5.49	0.63
5Cu/SiO_2_-AE	231	0.81	9.9	4.98	27.94	4.74
5Cu/SiO_2_-AE-C300	229	0.56	8.7	4.89	26.35	3.15
5Cu/SiO_2_-AE-C500	221	0.54	8.5	4.91	21.34	3.22
5Cu/SiO_2_-AE-R200	227	0.63	9.2	4.97	-	-
5Cu/SiO_2_-AE-R300	213	0.61	10.1	4.95	15.47	2.37
5Cu/SiO_2_-AE-R350	215	0.60	9.6	4.93	12.06	1.67

*^a^* Obtained using the BET method. *^b^* Cu metal particle size determined using Cu dispersion. *^c^* Determined via ICP-OES. *^d^* Cu dispersion was determined via N_2_O surface oxidation followed by TPR. *^e^* Cu metal surface area based on an atomic surface density of 1.46 × 10^19^ Cu atoms m^−2^.

**Table 2:** Catalytic hydrogenation of FAL to FOL with Cu/SiO_2_-AE catalysts.

Entry	Catalysts	FAL Conversion (%)	FOL Selectivity (%)	TOF (h^−1^)
1 *^a^*	5Cu/SiO_2_-AE	1.7	>99.9	-
2	5Cu/SiO_2_-AE-C300	19.7	98.3	15.9
3	5Cu/SiO_2_-AE	55.2	>99.9	36.0
4	5Cu/SiO_2_-AE-C500	23.1	>99.9	26.7
5	5Cu/SiO_2_-AE-R200	6.1	>99.9	-
6	5Cu/SiO_2_-AE-R300	24.6	>99.9	30.5
7	5Cu/SiO_2_-AE-R350	22.8	>99.9	31.4

*^a^* P_(N2)_ = 1 MPa. Reaction conditions: T = 90 °C, P_(H2)_ = 1.0 MPa, and t = 2 h.

## Data Availability

Data will be made available on request.

## Supplementary Materials

The following supporting information can be downloaded at: https://www.mdpi.com/article/10.3390/molecules30020225/s1, Figure S1: N_2_ adsorption-desorption isotherms and pore size distributions of the catalysts at different calcination temperatures; Figure S2: N_2_ adsorption-desorption isotherms and pore size distributions of the catalysts at different reduction temperatures; Figure S3: XRD patterns at different reduction temperatures of Cu/SiO_2_-AE; Figure S4: TEM images of catalysts; Figure S5: Cu 2p XPS and Cu LMM auger spectra of Cu/SiO_2_-AE at different calcination temperatures; Figure S6: Stability and TG on 5Cu/SiO_2_-AE; Figure S7: TEM images on used 5Cu/SiO_2_-AE; Figure S8: in situ FT-IR spectra of FAL desorption on the catalysts; Figure S9: in situ FT-IR spectra of FAL desorption on 5Cu/SiO_2_-IWI; Figure S10: in situ FT-IR spectra of FAL desorption by temperature program on 5Cu/SiO_2_-IWI; Table S1: Surface Cu components of 5Cu/SiO_2_-IWI and Cu/SiO_2_-AE based on Cu 2p and Cu LMM deconvolution; Table S2: The ratio of I_674_/I_800_ on catalysts.
