# Supplementary material for: Refining Surface Copper Species on Cu/SiO2 Catalysts to Boost Furfural Hydrogenation to Furfuryl Alcohol
Source: Molecules. 2025 Jan 8;30(2):225. doi: 10.3390/molecules30020225 (PMC11767369; doi:10.3390/molecules30020225)
Supplement: Supplementary file 1 [file molecules-30-00225-s001.zip › molecules-3370885-supplementary.pdf]

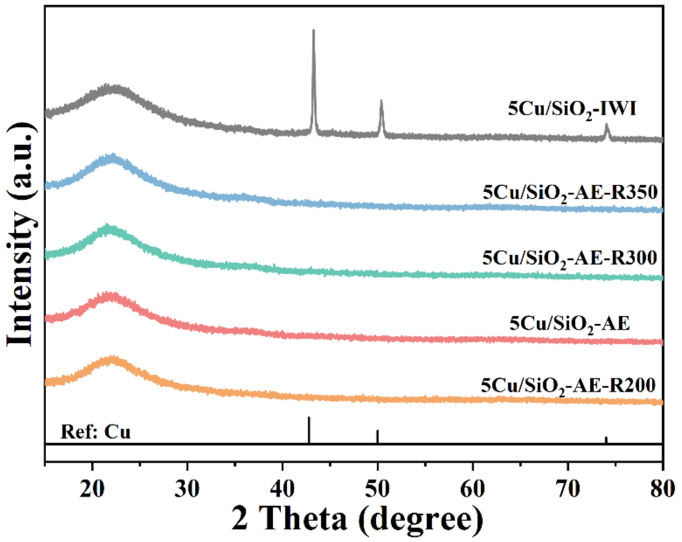

Figure S1. XRD patterns at different reduction temperatures of Cu/SiO_2-AE.

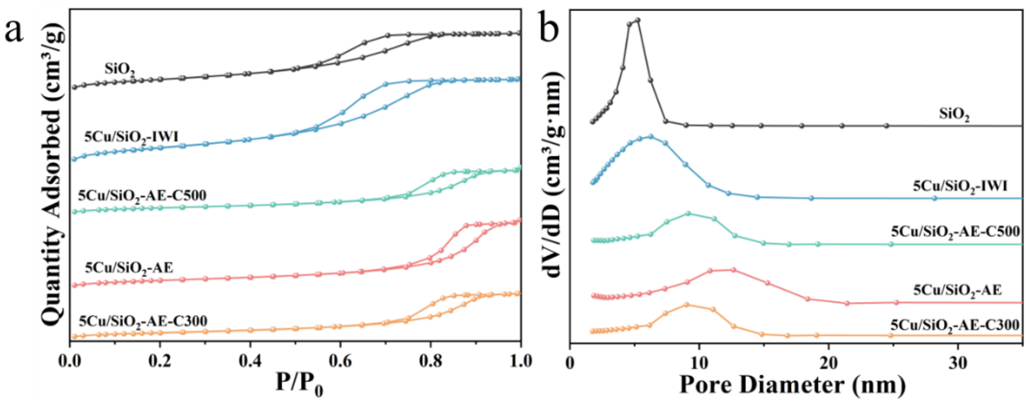

Figure S2. (a)N_2 adsorption-desorption isotherms and (b) pore size distributions of the catalysts at different calcination temperatures.

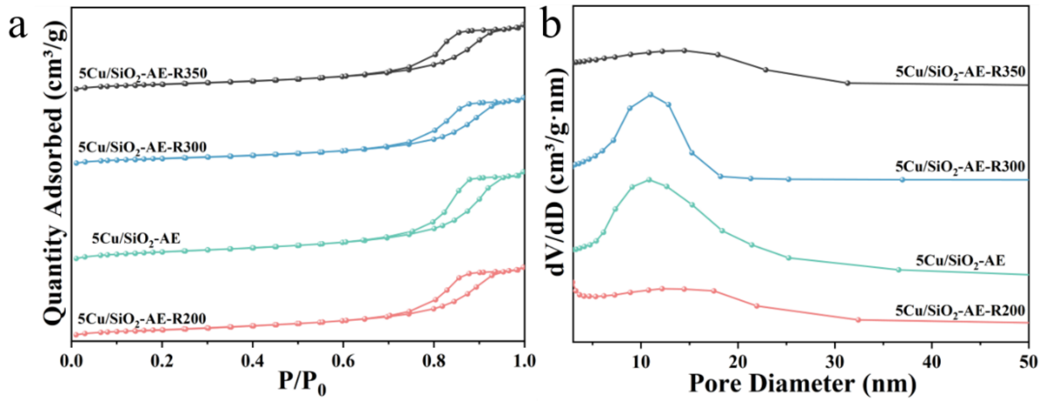

Figure S3. (a)N_2 adsorption-desorption isotherms and (b) pore size distributions of the catalysts at different reduction temperatures.

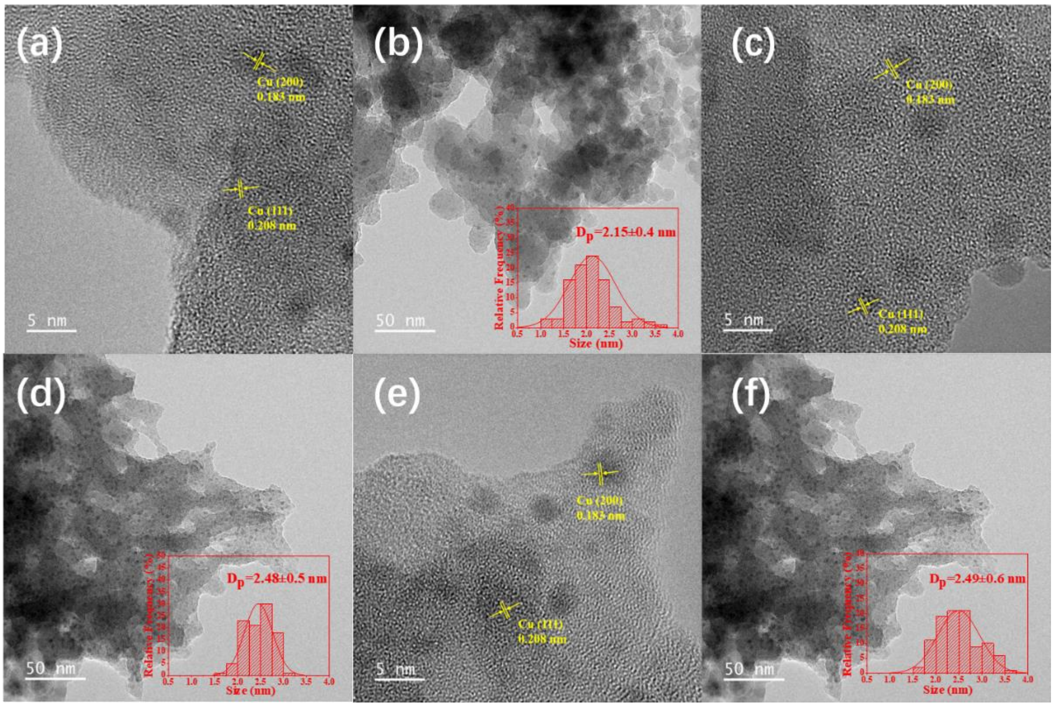

Figure S4. TEM images of catalysts: (a-b) 5Cu/SiO_2-AE-R200, (c-d) 5Cu/SiO_2-AE-R300, (e-f) 5Cu/SiO_2-AE-R350.

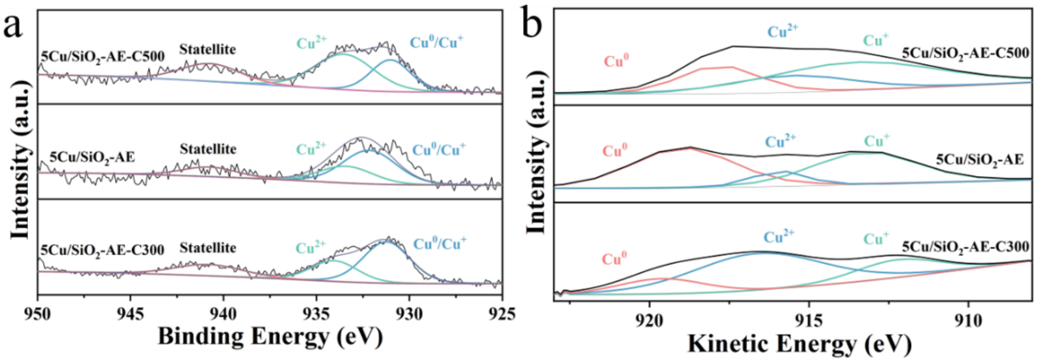

Figure S5. (a) Cu 2p XPS and (b) Cu LMM auger spectra of Cu/SiO_2-AE at different calcination temperatures.

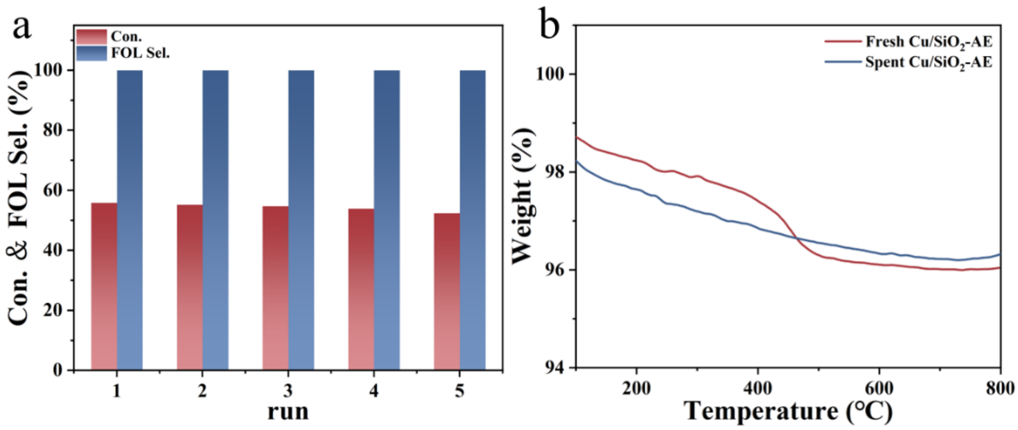

Figure S6. (a)Stability and (b)TG on 5Cu/SiO_2-AE

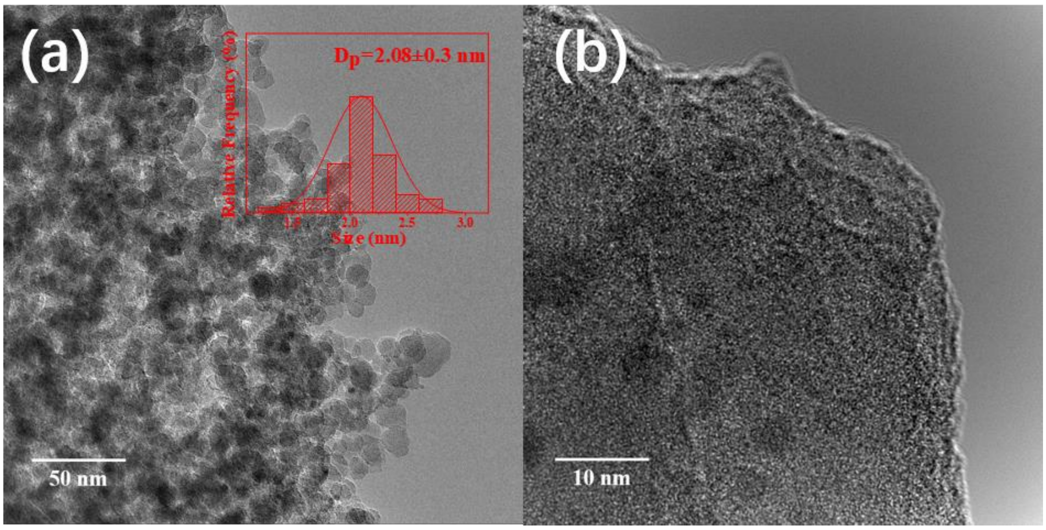

Figure S7. TEM images on used 5Cu/SiO_2-AE

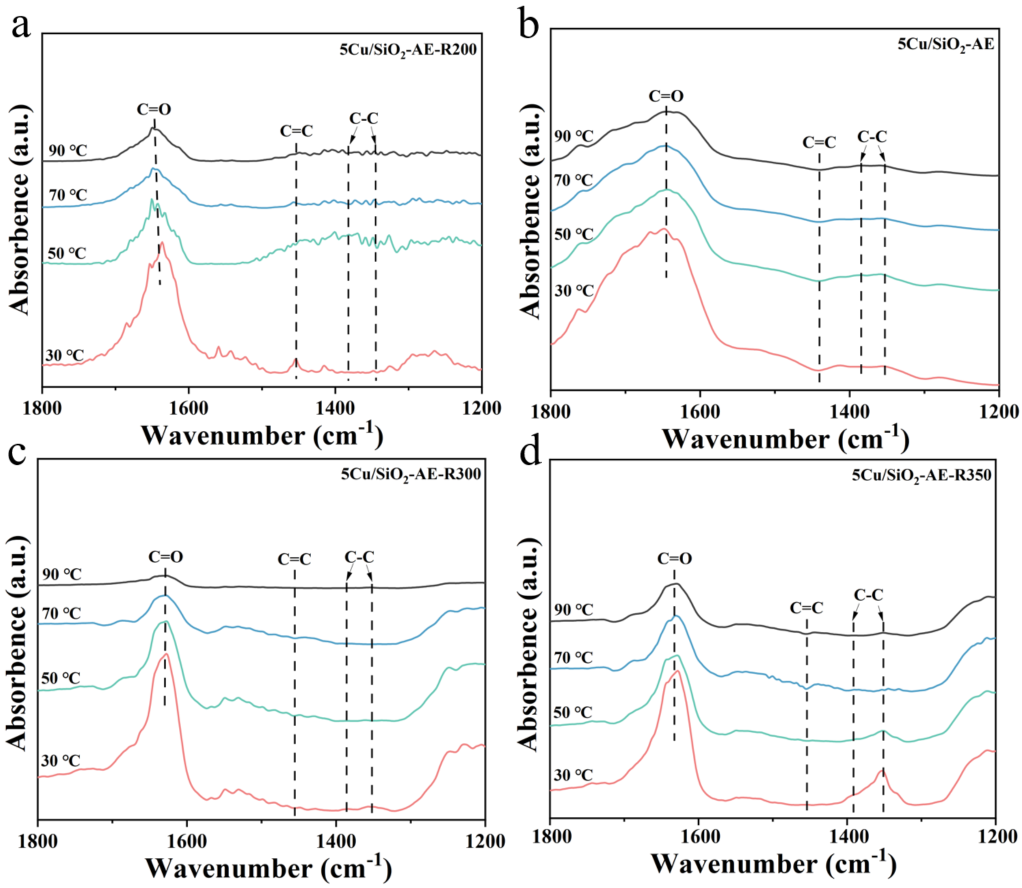

Figure S8. *in situ* FT-IR spectra of FAL desorption on the catalysts.

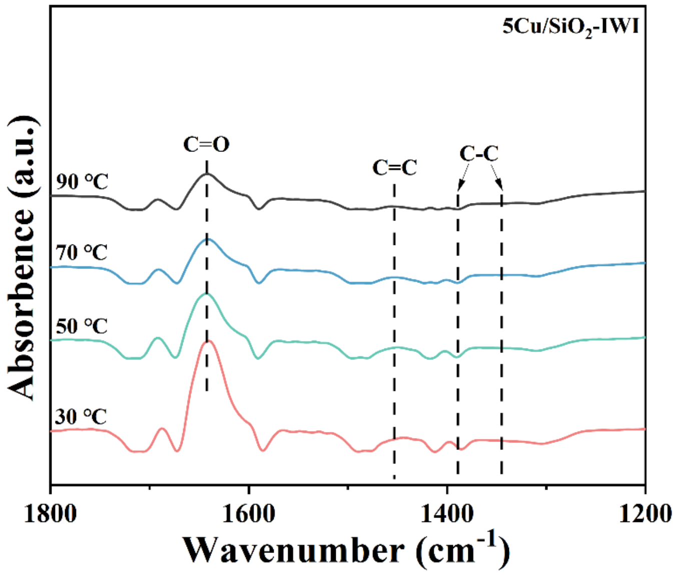

Figure S9. *in situ* FT-IR spectra of FAL desorption on 5Cu/SiO_2-IWI.

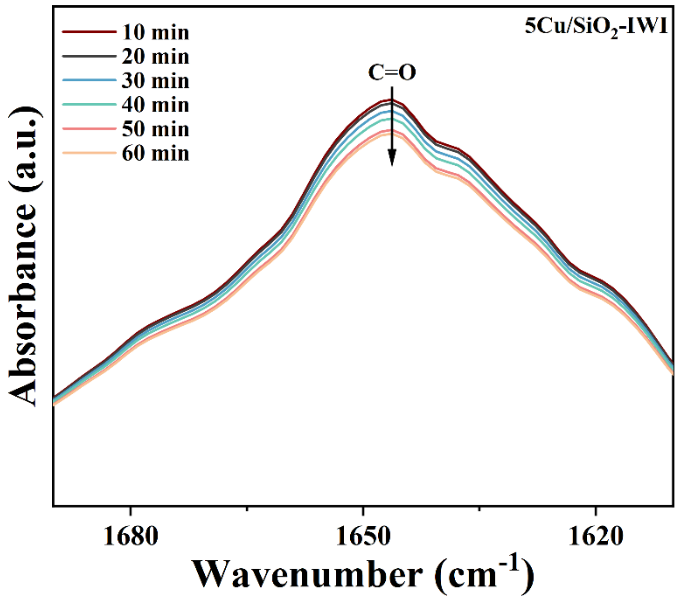

Figure S10. *in situ* FT-IR spectra of FAL desorption by temperature program on 5Cu/SiO_2-IWI.

Table S1. Surface Cu components of 5Cu/SiO_2-IWI and Cu/SiO_2-AE based on Cu 2p and Cu LMM deconvolution.

Catalyst	$(Cu^0+Cu^+)/Cu$ [a] (%)	$Cu^+/(Cu^0+Cu^+)$ [b] (%)	$Cu^0/(Cu^0+Cu^+)$ [b] (%)
5Cu/SiO_2-IWI	66.7	16.0	84.0
5Cu/SiO_2-AE	66.7	45.4	54.6
5Cu/SiO_2-AE-C300	62.7	17.4	20.6
5Cu/SiO_2-AE-C500	37.5	52.8	24.5
5Cu/SiO_2-AE-R200	33.3	35.6	64.4
5Cu/SiO_2-AE-R300	66.7	27.4	72.6
5Cu/SiO_2-AE-R350	66.7	19.2	80.8

[a] $(Cu^0+Cu^+)/Cu$ calculated from Cu 2p XPS spectra.

[b] $Cu^+/(Cu^0+Cu^+)$ calculated from Cu LMM XAES spectra.

Table S2. The ratio of I_{674} / I_{800} on catalysts.

Catalysts	I_{674}/I_{800} [a]
5Cu/SiO_2-IWI	-
5Cu/SiO_2-AE	0.19
5Cu/SiO_2-AE-C300	0.13
5Cu/SiO_2-AE-C500	0.02

[a] By integration of the fitted peaks at 674 cm^{-1} and 800 cm^{-1} in the FT-IR spectrum.
